# Contact angle and stability of interfacial nanobubble supported by gas monolayer

**DOI:** 10.1016/j.fmre.2022.05.005

**Published:** 2022-05-14

**Authors:** Haichang Yang, Yaowen Xing, Fanfan Zhang, Xiahui Gui, Yijun Cao

**Affiliations:** a Chinese National Engineering Research Center of Coal Preparation and Purification, China University of Mining and Technology, Xuzhou 221116, China; b School of Chemical Engineering and Technology, China University of Mining and Technology, Xuzhou 221116, China; c Henan Province Industrial Technology Research Institution of Resources and Materials, Zhengzhou University, Zhengzhou 450001, China

**Keywords:** Interfacial tension, Hydrophobicity, Potential of mean force, Gas density, Molecular dynamics simulations

## Abstract

Since solid-liquid interfacial nanobubbles (INBs) were first imaged, their long-term stability and large contact angle have been perplexing scientists. This study aimed to investigate the influence of internal gas density and external gas monolayers on the contact angle and stability of INB using molecular dynamics simulations. First, the contact angle of a water droplet was simulated at different nitrogen densities. The results showed that the contact angle increased sharply with an increase in nitrogen density, which was mainly caused by the decrease in solid-gas interfacial tension. However, when the nitrogen density reached 2.57 nm^−3^, an intervening gas monolayer (GML) was formed between the solid and water. After the formation of GML, the contact angle slightly increased with increasing gas density. The contact angle increased to 180° when the nitrogen density reached 11.38 nm^−3^, indicating that INBs transformed into a gas layer when they were too small. For substrates with different hydrophobicities, the contact angle after the formation of GML was always larger than 140° and it was weakly correlated with substrate hydrophobicity. The increase in contact angle with gas density represents the evolution of contact angle from macro- to nano-bubble, while the formation of GML may correspond to stable INBs. The potential of mean force curves demonstrated that the substrate with GML could attract gas molecules from a longer distance without the existence of a potential barrier compared with the bare substrate, indicating the potential of GML to act as a gas-collecting panel. Further research indicated that GML can function as a channel to transport gas molecules to INBs, which favors stability of INBs. This research may shed new light on the mechanisms underlying abnormal contact angle and long-term stability of INBs.

## Highlights

• Evolutionary of contact angle of bubble from macroscale to nanoscale is researched.
• GML coexists with bubble only when internal gas density is high.
• Contact angle is large regardless of surface hydrophobicity after formation of GML.
• GML attracts gas molecules from bulk and assists the stability of interfacial nanobubble.

## Graphical abstract

**Figure:**
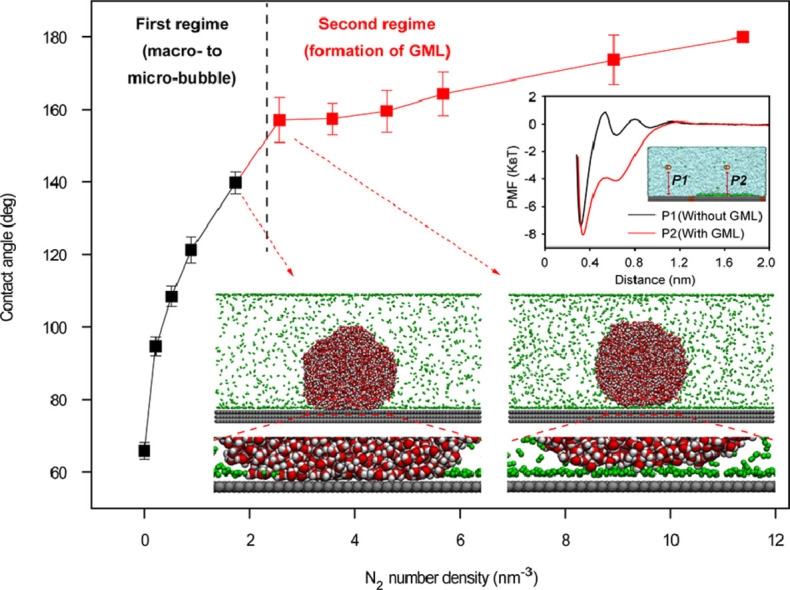

## 1 Introduction

Interfacial nanobubbles (INB), usually defined as nanoscopic gaseous spherical caps, were first introduced by Parker et al. [1] in 1994 as the potential dominant factor causing long-range attractive forces between two approaching hydrophobic surfaces in an aqueous solution. However, the long-term existence of INB was widely doubted in the early stages, because INB was expected to dissolve within milliseconds owing to the ultrahigh Laplace pressure according to classical physical theory [2]. This opinion was held even after the first INB image was obtained using atomic force microscopy (AFM) in 2000 [3,4]. Nevertheless, with the gradual detection of INB using many techniques, such as AFM [3], attenuated total reflectance infrared spectroscopy [5], surface plasmon resonance [5], and neutron reflectometry [6], its existence is now widely accepted by the scientific community [7], [8], [9], [10]. However, the unique properties of INBs, such as long lifetime and large contact angle (from the liquid side), remain a challenge for scientists to explain.

Brenner and Lohse [11] in 2008 proposed a dynamic equilibrium theory to explain INB stability. They suggested that the gas outflux from INBs driven by Laplace pressure could be compensated for by gas influx through the contact line. Their model could explain the experimental results on electrolytically generated INBs [12], but for INBs generated by other methods, they could neither find the energy source driving such gas circulation nor find experimental evidence [13,14]. A year later, Ducker [15] proposed the contamination theory. He suggested that contamination could reduce the surface tension of the liquid as well as hinder gas diffusion outward from the INB, and these effects become stronger with the shrinkage of INB due to the concentration of contaminants, thus producing negative feedback to resist further shrinking of INB. However, this theory fails to explain the fact that INBs are still stable when organic contaminants are removed from gas-water interface using surfactants above critical micelle concentration [16]. Furthermore, gas-water interface of INBs was found to be permeable [17]. More recently, Lohse and Zhang [18] developed a pinning-oversaturation model based on the modified Epstein-Plesset theory, suggesting that pinning, gas oversaturation, and hydrophobic substrates guarantee the stability of INBs considering that contact line pinning widely exists and that gas oversaturation is always the requirement for producing INBs. For a pinned INB, its curvature radius and contact angle will increase with the shrinking of INB; thus, the Laplace pressure decreases, forming negative feedback to stabilize the INBs. However, experimental observations have shown that INBs still exist when they are depinned [19,20], in undersaturated aqueous solutions [21], or on hydrophilic surfaces [20]. To resolve these discrepancies, Tan et al. [22,23] introduced hydrophobic attraction between hydrophobic substrate and air molecules into the pinning-oversaturation theory to guarantee a localized oversaturation environment near the substrate, even in an undersaturated solution. Tan's model successfully explains the stability of INBs under ambient and degassing conditions; however, contact line pinning and hydrophobic substrates are mandatory. Thus, a convincing overarching theory has not been fully developed, and further research is required.

In addition to its long lifetime, another anomalous feature of INB is that its contact angle (usually larger than 140°) is far larger than that of its macroscopic counterpart [8]. A higher contact angle of INB signifies a larger curvature radius for a given lateral diameter, thus, a smaller Laplace pressure; hence, a higher contact angle could prolong the lifetime of the INB. Therefore, uncovering the mechanism behind the large contact angle of INB may be helpful in understanding its stability. Contact line pinning [24] can increase the contact angle through contact line hysteresis caused by surface roughness or chemical heterogeneity during INB shrinkage; however, it is difficult to account for the large increase in contact angle as contact angle hysteresis is usually lower than 30° for a smooth substrate; in addition, it cannot explain the stability of unpinned INBs [19,20]. The effect of line tension was used to explain the large difference between the nano- and macro-scopic contact angles. However, researchers have always obtained a line tension of ∼ 10^−10^ N by fitting experimental data, which is much larger than the theoretical value of ∼ - (10^−11^ −10^−12^) N [8]. In addition, the line tension would turn to decreasing contact angle when contact angle is larger than 90° [25]. Weijs et al. [26] demonstrated that the increase in gas density near the substrate outside INB should be the origin of large contact angle of nanobubble, because the presence of gas weakens the solid-liquid interaction and renders the substrate more hydrophobic. Petsev et al. [27] used Szyszkowski equation to explain the influence of gas density inside INB on contact angle. They suggested that the solid-gas interfacial energy decreases with an increase in internal gas density of INB, and thus, the contact angle is increased according to Young's equation. According to their theory, the contact angles of both hydrophobic and hydrophilic substrates can increase significantly for a nanoscale bubble, which we think is a significant mechanism for large contact angle of INB. However, this theory could not explain why the contact angles of INBs are weakly correlated with substrate hydrophobicity.

Wang et al. [28] found a high-density gas state inside INB through molecular dynamics (MD) simulations in 2008. Recently, Zhou et al. [29] successfully found the experimental evidence for highly condensed gas state inside INBs with the help of synchrotron-based scanning transmission X-ray microscopy. They suggested that the oxygen density inside the bubble is approximately 76.3−123.7 times greater than that at atmospheric pressure, and the internal gas density increases with a decrease in INB's lateral diameter. Wang et al. [30] also revealed through force spectroscopy analysis that the high gas density layer inside INB should be three orders of magnitude larger than that at standard temperature and pressure. In addition to the high internal gas density, the existence of a gas layer outside INBs has been observed in AFM experiments [31], [32], [33], [34], [35], [36]. By using AFM with frequency-modulation mode (FM-AFM), Lu et al. [33] demonstrated that all highly oriented pyrolytic graphite (HOPG) surfaces in gas-supersaturated water are fully covered by GML, with some INBs resting on top. Peng et al. [36] found that the entire area between INBs was covered with a gas layer using a force mapping technique. Using high-resolution FM-AFM, Schlesinger et al. [34] suggested that a 2−5 nm gas layer spontaneously forms on a hydrophobic surface. However, the influence of the internal ultra-high gas density and external gas layer on the contact angle, as well as stability of INB, has not been fully understood.

In the current study, we examined the influence of gas density on the contact angle of INB, explored the formation condition of intervening GML between solid and water using molecular dynamics (MD) technique, and also evaluated the role of GML in contact angle and stability of INB. This study emphasizes on understanding as to why the contact angle of INB is much larger than its macroscopic corresponding angle, and why it weakly correlates with substrate hydrophobicity; it also elucidates the mechanism by which the GML supports INB stability. Our current work may shed new light on the mechanism underlying abnormal contact angle of INB, as well as its long-term stability.

## 2 Simulation methodology

All-atom MD simulations in the current study were performed using the open-source code GROMACS 2019.6 [37]. The simulation model was visualized using VMD 1.9.3 [38]. Interatomic interactions were modeled using a combination of Lennard-Jones (LJ) 6-12 potential and Coulomb potential:

(1) $$U\left(r_{ij}\right)=4\varepsilon_{ij}\left({\left(\frac{\sigma_{ij}}{r_{ij}}\right)}^{12}-{\left(\frac{\sigma_{ij}}{r_{ij}}\right)}^{6}\right)+f\frac{q_{i}q_{j}}{r_{ij}}\;$$

where $\varepsilon_{ij}$ is the LJ potential well depth; $\sigma_{ij}$ is the characteristic size between atoms *i* and *j*, separated by a distance $r_{ij}$; $q_{i}$ and $q_{j}$ are the charges of atoms *i* and *j*, respectively; and the electric conversion factor $f=1/\left(4\pi \in_{0}\right)=138.935458\;\text{kJ}\;\text{mo}l^{-1}\;\text{nm}\;e^{-2}$, where $\in_{0}$ is the permittivity of free space. Periodic boundary conditions were set in all three directions to avoid problems with boundary effects caused by finite size. A widely used SPC/*E* water model was employed to simulate the liquid. A two-site nitrogen model was used for the gas [39], and three layers of graphene (bond length = 0.42 nm) were used for the solid. The interaction parameters for the liquid and gas are listed in Table 1. For cross-interaction we used *σ_lg_* = 0.3213 nm and *ε_lg_* = 0.4333 kJ/mol, while the solid-gas and solid-liquid interaction parameters are listed in Table 2.

**Table 1:** **LJ parameters of the different atoms used in simulation**.

Atom	σ(nm)	ε(kJ/mol)	Charge (e)
N (Nitrogen)	0.3261	0.2887	0
O_w_ (water)	0.3166	0.6502	-0.8476
H_w_ (water)	0	0	0.4238

**Table 2:** LJ parameters.

Substrates	SG_SL	SG_SL1	SG_SL2	SG_SL3	SG1_SL	SG2_SL	SG3_SL	SG4_SL	SG5_SL
$\varepsilon_{sg}\left(kJ/mol\right)$	0.6500	0.6500	0.6500	0.6500	0.3364	0.4400	0.5500	0.7500	1.0000
$\varepsilon_{sl}\left(kJ/mol\right)$	0.5049	0.6100	0.4000	0.3200	0.5049	0.5049	0.5049	0.5049	0.5049

Note: (*σ_sl_, σ_sg_*) = (0.3178, 0.3225) nm for all the simulations. Subscript of *s, l* and *g* represent the C atom of the graphene substrate, O atom of water, and N atom of nitrogen, respectively. SG-serial substrates, such as SG_SL, SG_SL1, SG_SL2 and SG_SL3, have the same solid-gas interaction parameters but different solid-liquid potential well, while SL-serials, such as SG_SL, SG1_SL, SG2_SL, SG3_SL, SG4_SL and SG5_SL, have the same solid-liquid interaction parameters but different solid-gas potential well.

The simulations for water droplets under vacuum or high gas density were run in a canonical (NVT, fixed number of atoms, *N*, volume, *V*, and temperature, *T*) ensemble, while those for INB simulation were run in a semi-isotropic constant-temperature, constant-pressure (NP_z_T) ensemble where the box was only scaled in the *z* direction, and the pressure in the z direction was kept constant at 1 bar using Parrinello-Rahman pressure coupling. V-rescale temperature coupling was applied to gas and water molecules to maintain a constant temperature of 300 K. A leapfrog integrator with a time step of Δt =2 fs was used throughout the simulations. The coulomb type was fast smooth particle-mesh Ewald electrostatics (PME), where the direct space was similar to the Ewald sum, while the reciprocal part was performed with FFTs. Both the cutoff radii for LJ interactions and real space of electrostatic interactions were 1.0 nm.

Cylindrical droplets/INBs were used to simulate contact angle since the line tension in this case was negligible because of the effective infinite length of the three-phase contact line considering periodic boundary condition [40]. The box size of the droplet simulation in the x, y, and z directions was 20.168 nm × 2.556 nm × 10.000 nm, while that for the INB simulation was 60.504 nm × 2.556 nm × 10.000 nm. For simulations of different gas densities, a certain number of gas molecules was added to obtain the desired gas density, and the number of water molecules was kept constant. The simulation time was 20 ns, unless otherwise stated. The contact angle was calculated once for each 0.1 ns of the simulation, and thus 200 contact angles were obtained during the entire 20 ns simulation. We found that all the simulations in this study reached equilibrium within 10 ns; hence, the average of the last 100 values (from the last 10 ns) was used for analysis.

The procedures for calculating the contact angles are as follows: First, the simulation box was cut into bins size of 0.1 nm × 2.556 nm × 0.1 nm. Second, the average density of the water molecules within each bin in each 0.1 ns was calculated. Third, the positions of the gas-water interface, namely, the bins with water densities equal to half of the bulk density were found. Fourth, the coordinates of these eligible bins were fitted with a circle function using the least-squares method to calculate the contact angle. Here, the mass center of the top solid layer plus $\sigma_{sl}/2$ is defined as the solid-liquid interface. For the cases in which a GML existed between the water and solid, z coordinate of the peak density of this dense gas layer plus $\sigma_{lg}/2$ was used as the interface to calculate contact angles. Only positions at least 0.4 nm above the interface were used for fitting to avoid the influence of density fluctuations near the interface.

We calculated the potential of mean force (PMF) along two paths (P1 and P2, Fig. 4) to investigate the influence of GML on adsorption of nitrogen molecules on the substrate. To guarantee the comparability of these two paths, they were simulated in one system, namely, a part of the substrate with GML and the other part without GML, which were separated by the modified atoms of weak aerophily. The procedure is described as follows: First, two single N_2_ molecules were pulled respectively towards the substrate along the two paths at a speed of 0.5 nm/ns from the initial normal distance of about 2.0 nm between N_2_ molecule and the top layer of graphene. Second, a series of configurations at an interval of 0.1 nm were generated along this reaction coordinate. Third, these configurations were simulated separately, and umbrella sampling was used to restrain these configurations within the sampling windows. For each umbrella sampling simulation, an equilibrium run of 2 ns was first performed, followed by a production run of 18 ns for sampling. The output data generated by umbrella sampling simulations were calculated using the weighted histogram analysis method (WHAM) [41] to construct PMF along these two paths.

## 3 Results and discussion

### 3.1 Evolution of contact angle with gas density

**Fig. 1:**
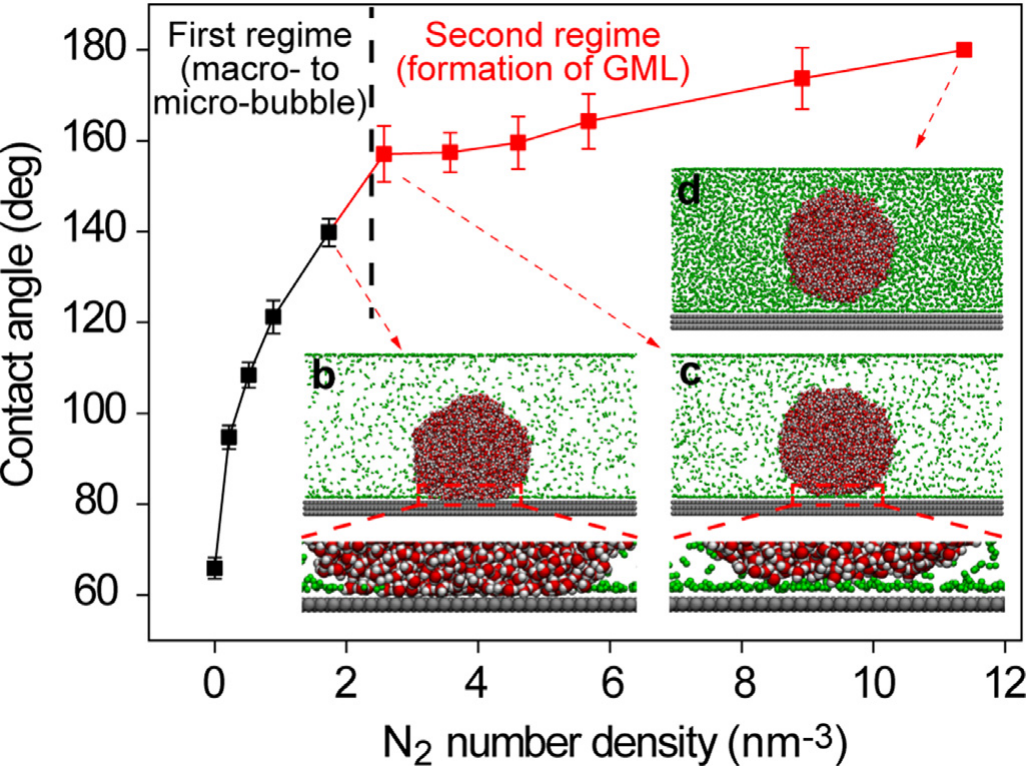
**Contact angle as a function of gas density for SG_SL substrate**. The insets are the snapshots of simulation. The red data marker represents the formation of GML. For simulation snapshot, the silver and green beads respectively represent solid and gas atoms, and the red and white beads respectively represent oxygen and hydrogen atoms of water molecule. The snapshots for all the simulations are shown in Fig. S3. The evolution of contact angle with time is shown in Fig. S4 which demonstrates the simulation had reached equilibrium within the first 10 ns.

To understand the influence of gas density on contact angle, we investigated the contact angle of a water droplet which was surrounded by different gas densities, and the results are shown in Fig. 1a . The contact angle of the water droplet under vacuum was 65.89° ± 2.33°, which is within the normal range because a freshly cleaved HOPG usually has a water contact angle of 60°−70° [34,42,43]. When the water droplet was placed in a high-density gas environment, the contact angle increased with gas density, as shown in Fig. 1a, but two obviously different regimes were observed. The first regime exhibited a steep increase in the contact angle with gas density, while the second regime exhibited a gas density larger than 2.57 nm^−3^, where the increasing trend was relatively gentle and an intervening GML between the water and solid was formed.

The sharp increase in the contact angle in the first regime was mainly due to the decrease in solid-gas interfacial tension. Petsev et al. [27] reported that the adsorption of gas molecules on a solid surface lowers the energy of the solid-gas interface, and quantifies them using the following equation:

(2) $$\gamma_{sg}^{*}=\gamma_{sg}-\frac{k_{B}T}{b}\ln\left(1+K_{eq}^{A}P\right)$$

where $\gamma_{sg}^{*}$ and $\gamma_{sg}$ are the solid-gas interfacial tensions at air pressure *P* and in vacuum; $k_{B}$ is the Boltzmann constant; T is the temperature; b is the cross-sectional area of an adsorbing gas molecule; and $K_{eq}^{A}$ is the equilibrium adsorption constant. The data was fitted in the first regime (black-color-marked data) in Fig. 1a using Eq. 2 with b and $K_{eq}^{A}$ as the fitting variables. The fitting process and results are presented in Supplementary Note 1. The goodness of fit to the experimental data is *R*^2^ = 0.9995, indicating an excellent description of the first regime in terms of Petsev et al. theory. We obtained $K_{eq}^{A}$ = 3.28 × 10^−6^ Pa^−1^, which is within the actual range of $K_{eq}^{A}$ = (1.0 ∼ 5.0) × 10^−6^ Pa^−1^ for N_2_, O_2_ and Ar adsorbing to graphene [27,44].

For the second regime in Fig. 1a, namely, a gas density ≥ 2.57 nm^−3^, an intervening GML between water and the substrate appears, as clearly shown in Fig. 1c; thus, the solid-liquid interface was replaced by a solid-gas interface and a liquid-gas interface. Although Eq. 2 describes the increase in contact angle with gas density in the first regime, the formation of a GML between water and the substrate at higher gas density in the second regime is not expected. The relationship between the contact angle and gas density in Fig. 1 may also be applied to a system of surface bubbles in water, as the contact angle of the bubble should be similar to that of a water droplet according to Young's equation. With decrease in bubble radius, the gas density inside the bubble increases owing to an increase in the Laplace pressure. Therefore, the increase in gas density corresponds to decrease in bubble size from macro- to micro- and to nano-scale. In the second regime, the existence of GML weakens the interaction between the solid and liquid, and makes the solid more hydrophobic; thus, the contact angle is extremely high in the second regime and is weakly related to the hydrophobicity of the substrate, which is similar to the case of a nanobubble. Hence, we speculate that the formation of GML in the second regime may correspond to stable INB. In the second regime, the increasing trend of the contact angle with gas density is relatively slow (red color in Fig. 1a). This is in agreement with the experimental findings that the contact angle of INB increases slightly with decrease in their height [16,[45], [46], [47]]. When the nitrogen density was particularly high, such as 11.38 nm^−3^ in Fig. 1d, the contact angle became 180°. This indicates that INBs would no longer exist when they are very small (except for INBs in confined areas such as nanoelectrodes), because the extremely high density of gas inside the tiny INB would transform itself into a GML. In contrast, if the bubble is large such that the internal gas density is relatively low, the GML would not form, and thus a stable INB would not exist if GML indeed accounts for the stability of INB. This could explain why INBs always have a preferential size range, dozens of nanometers to several micrometers in width, which might be the range in which INBs could coexist with GML. The change in contact angle with gas density shown in Fig. 1a represents the evolution of contact angle from macro-bubble to nano-bubble, but the transitory stage before the formation of GML is difficult to observe in experiments, because the bubble shrinks rapidly according to the Epstein-Plesset theory [48].

**Fig. 2:**
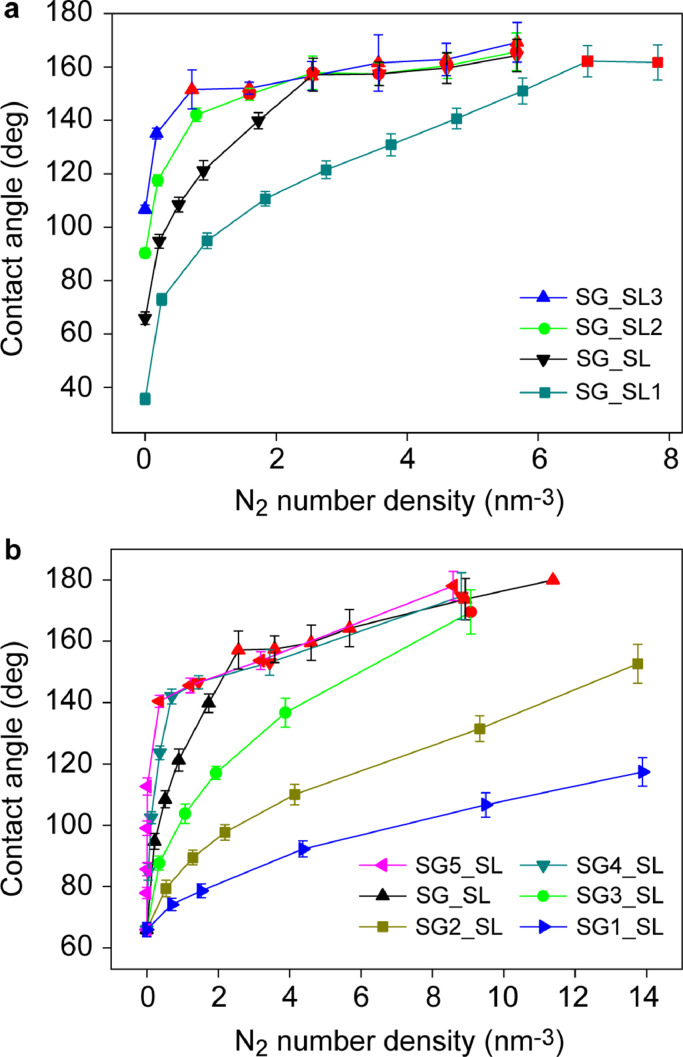
**Contact angle as a function of gas density for different substrates**. (a) Substrates with the same solid-gas interaction but with the different solid-liquid interaction; (b) substrates with the same solid-liquid interaction but with the different solid-gas interaction. Note: the red color indicates the formation of GML; the detailed parameters of these substrates are shown in Table 2.

To investigate the influence of gas density on contact angle for substrates with different hydrophobicities, the contact angles of a water droplet standing on substrates of SL1, SL, SL2, and SL3 were simulated; the results are shown in Fig. 2a. The contact angles for SL1, SL, SL2, and SL3 in vacuum are 35.71° ± 2.12°, 65.89° ± 2.33°, 90.30° ± 1.81° and 106.72° ± 1.52°, respectively. For all these substrates, the contact angle increased steeply with increasing gas density, and then increased gradually after the formation of GML. From the red marker in Fig. 2a, we found that the contact angles after the formation of GML were very high, mainly in the range of 140°−180° regardless of surface hydrophobicity, which is consistent with previous experimental observations that the contact angles of INBs are ultrahigh and weakly related to substrate hydrophobicity [8,[49], [50], [51]]. After the formation of GML, water molecules contacted immediately with gas molecules rather than the substrate, and the interaction between water molecules and the substrate could not be completely blocked by GML; hence, the contact angle was weakly related to the substrate hydrophobicity.

We then investigated the relationship between contact angle and nitrogen density for substrates with different solid-gas interactions; the results are shown in Fig. 2b. The contact angle increases with increasing gas density for all substrates, but this trend is sharper for substrates with higher $\varepsilon_{sg}$. This might be because the decrease in solid-gas interface energy with increasing gas density was more significant for substrates with higher $\varepsilon_{sg}$. The same method as mentioned in Supplementary Note 1 was used to calculate solid-gas equilibrium adsorption constant $K_{eq}^{A}$, and the results showed that the values of $K_{eq}^{A}$ for SG1, SG2, SG3, SG, SG4, and SG5 were 1.2 × 10^−7^, 3.2 × 10^−7^, 1.2 × 10^−6^, 3.3 × 10^−6^, 1.0 × 10^−5^, and 1.4 × 10^−4^ Pa^−1^ respectively. According to Eq. 2, the higher the $K_{eq}^{A}$, the faster the increase in $\varepsilon_{sg}$ with gas density. The value of $K_{eq}^{A}$ between different solid and gas types usually range from 10^−6^ to 10^−3^ Pa^−1^; hence SG1, SG2, SG3, SG, SG4, and SG5 in Fig. 2b represent different solid and gas types with different equilibrium adsorption constants. GML formation was observed on substrates SG_SL, SG3_SL, SG4_SL, and SG5_SL. The common condition of these four substrates is $\varepsilon_{sg}>\varepsilon_{sl}$, which is typically used with a hydrophobic substrate in MD simulations [52,53]. Therefore, $\varepsilon_{sg}>\varepsilon_{sl}$ might be a precondition for the formation of GML. This is always true for hydrophobic surfaces due to weak solid-liquid interactions, and hence it is easier to form GML and thus stable INBs. However, for solids with $\varepsilon_{sg}<\varepsilon_{sl}$, such as substrates of SG1_SL and SG2_SL, GML could not form even when the gas density reached as high as 14 nm^−3^.

### 3.2 Formation of GML outside INB

**Fig. 3:**
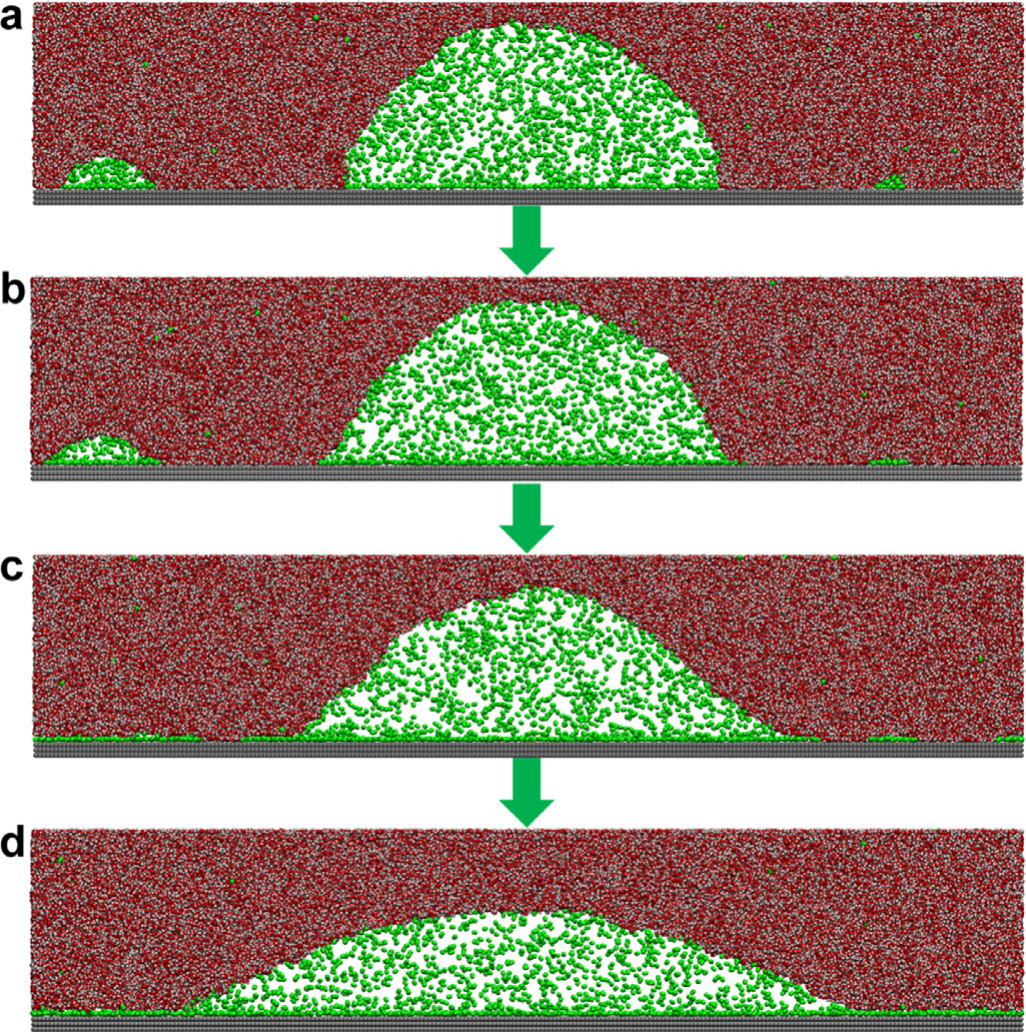
**The evolutionary process of INB on SG_SL substrate**. (a) Initial configuration; (b) snapshot of 60 ps; (c) snapshot of 800 ps; (d) snapshot of 20 ns. The evolution of contact angle with time is shown in Fig. S6, which shows the simulation reached equilibrium within 10 ns and kept stable in the following 10 ns.

Next, we simulated the INB system as shown in Fig. 3. The box size was 60.504 nm × 2.556 nm × 10.000 nm. The initial configuration was obtained by simulating on SG1_SL substrate for 10 ns using 2000 nitrogen molecules and 42862 water molecules, as shown in Fig. 3a; where three INBs with different sizes were attached on the substrate without the formation of GML, which accords with Fig. 2b that GML could not form on SG1_SL. The substrate was then replaced with SG_SL. It was found that the smallest nanobubble quickly turned into a GML (Fig. 3b), followed by the second smallest one (Fig. 3c). Meanwhile, the gas molecules inside the largest nanobubble also transformed into a GML until the surface was fully covered by the GML, after which a stable INB was formed (Fig. 3d). Owing to the high gas density and the existence of GML, the contact angle of INB (146.80°) is much larger than that of the water droplet on the SG_SL substrate (65.89°). When the hydrophobicity of the substrate changed (to SG_SL2 or SG_SL3), as shown in Fig. S5, INB still coexisted with GML and remained stable at a large contact angle. The contact angles of INB formed on SG_SL, SG_SL2, and SG_SL3 were 146.80° ± 0.88°, 148.83° ± 1.22° and 150.24° ± 1.00° respectively, while their vacuum contact angles were 65.89° ± 2.33°, 90.30° ± 1.81° and 106.72° ± 1.52°, respectively. This again demonstrates that the contact angle of INB weakly correlates with hydrophobicity of the substrate when GML is formed.

Here, we qualitatively analyzed the formation mechanism of GML. The gas layer can be regarded as a type of liquid, due to which the molecule distribution density in the gas layer (26 nm^−3^) reaches the order of that in liquid nitrogen (17.5 nm^−3^ at 77 *K*) (Fig. S7). Subsequently, we investigated the spreading of the gas layer from inside the bubble to the solid-water interface through the energy barrier, which is given by:

(3) $$\Delta G=A(\gamma_{s-layer}+\gamma_{l-layer}-\gamma_{sl})+\Delta \text{PV}$$

where *A* is the coverage area of GML; γ_s-layer_, γ_l-layer_, and γ_sl_ are the interfacial tensions of the solid, liquid and solid-liquid interfaces, respectively; and ΔPV is the change in pressure volume of INB due to the outflow of gas molecules. According to the Langmuir isotherm model [54], the density of the gas layer increases with increasing gas density, resulting in γ_s-layer_ and γ_l-layer_ decreasing with increasing gas density. Thus, when the gas density increases to a critical value that makes Δ*G* < 0, the gas layer spreads to the solid-liquid interface.

### 3.3 Stability mechanism of INBs based on GML

**Fig. 4:**
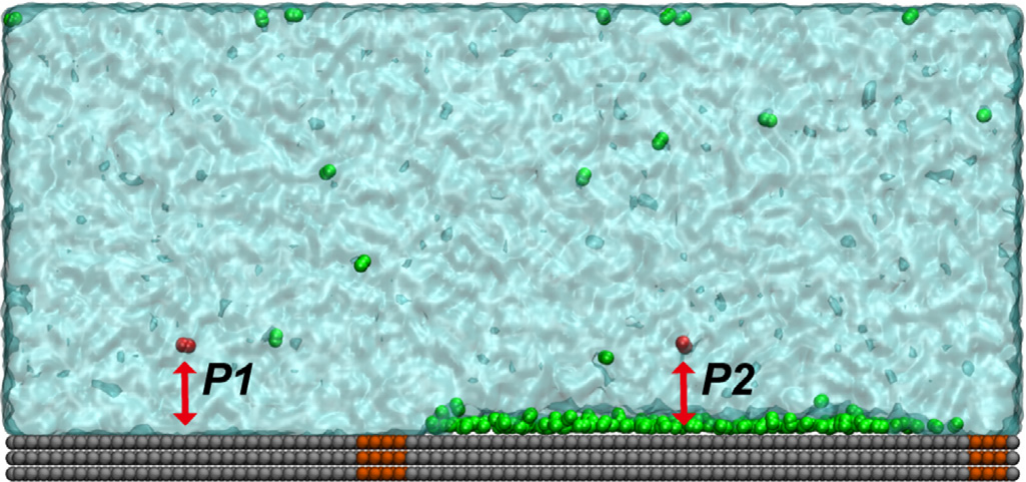
**The simulation system for calculating PMF of N_2_ molecule towards substrate with/without GML**. Note: the silver beads represent SG_SL substrate, the orange beads represent SG1_SL substrate to separate the substrate into two parts (left part without GML and right part with GML), the green beads represent nitrogen molecules, the red beads represent nitrogen molecules that were pulled towards the substrate, and the cyan represents water phase.

**Fig. 5:**
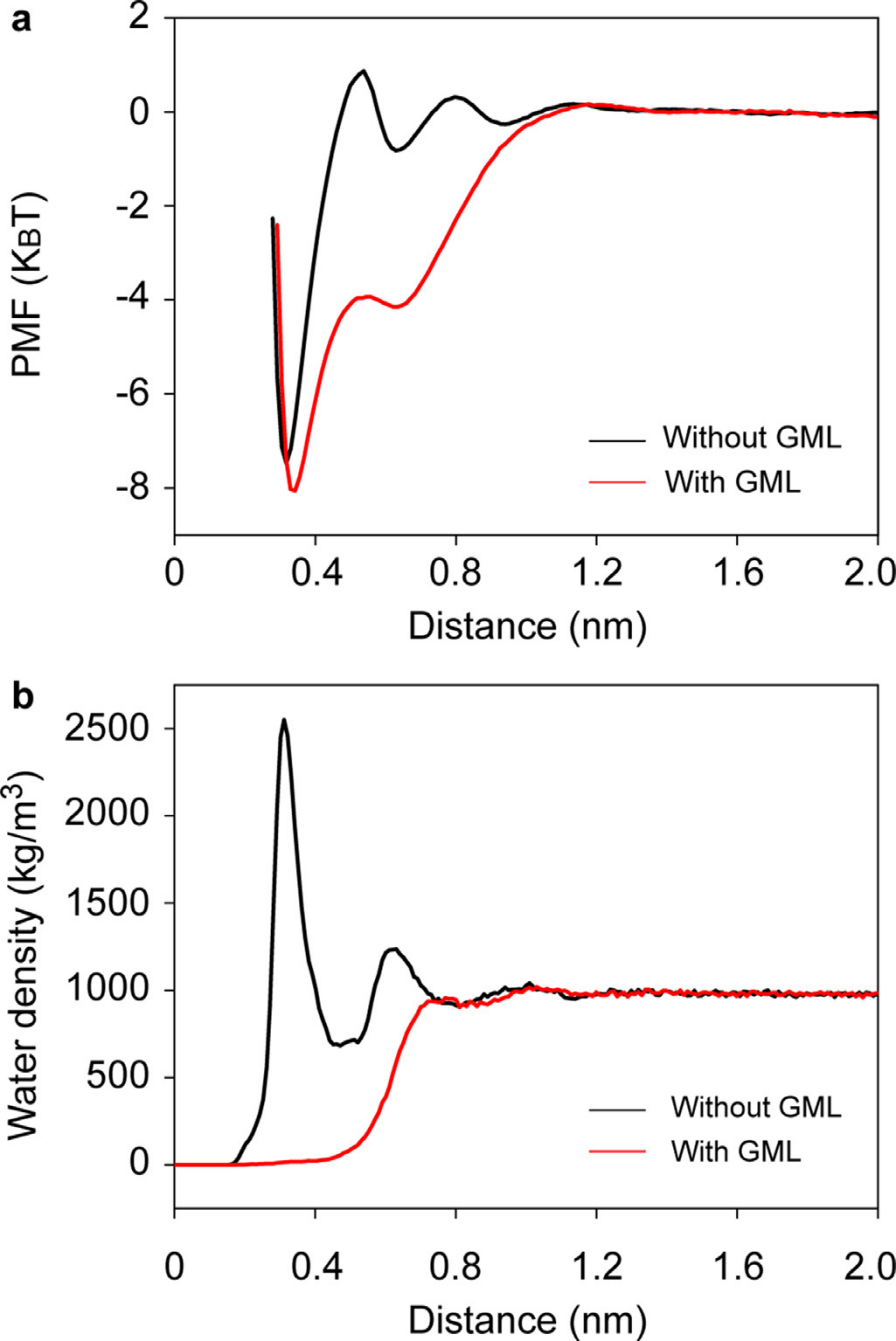
**The impact of GML on the interaction between substrate and dissolved gas molecules**. (a) PMF curves of N_2_ molecule towards substrate with/without GML; (b) the water density profile along the normal direction of solid-water interface with/without GML. Note: the distance of x-coordinate represents the normal distance from the top layer of substrate.

**Fig. 6:**
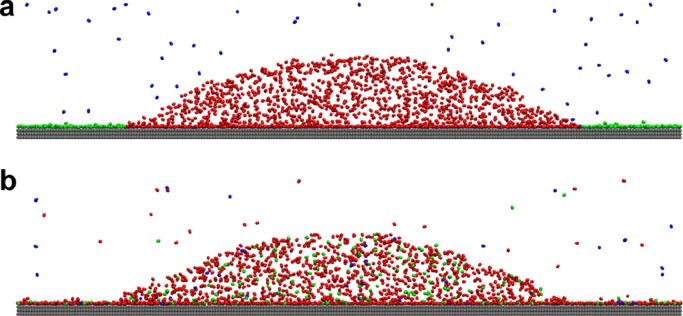
**The fluidity of N_2_ molecules in GML, INB and bulk liquid**. (a) The snapshot of simulation at the moment of 10 ns, red beads represent N_2_ molecules in INB, the green beads represent N_2_ molecules in GML, and the blue beads represent N_2_ molecules in bulk liquid; (b) these N_2_ molecules are intermingled with each other at the moment of 20 ns.

If INBs are stabilized by GML, the GML should be able to attract gas molecules from the bulk and transport these gas molecules to the inside of INBs. To verify this point, we calculated PMF of N_2_ molecules towards the substrate with/without GML. The simulation system is shown in Fig. 4 and the PMF curves are shown in Fig. 5a. The results show that there is a substantial potential well between nitrogen molecules and graphite substrates with/without a GML, but differences exist. For the bare substrate, the N_2_ molecule is attracted by the substrate from distance of 0.54 nm, while this critical distance for the substrate with GML is 1.18 nm. This indicates that the substrate with GML can attract gas molecules from a greater distance. In addition, there are two potential barriers of 0.31 and 0.87 k_B_T respectively when N_2_ molecules are diffusing towards the solid-liquid interface of the bare substrate. These barriers might be caused by the formation of interfacial hydrated layers on the bare HOPG surface, as shown in Fig. 5b, which is consistent with Schlesinger and Sivan [34], who reported that hydration of HOPG surface occurs despite its hydrophobic nature. However, for the substrate with GML, there is no hydration layer, as shown in Fig. 5b; hence, there is almost no potential barrier, as shown in Fig. 5a, which favors the adsorption of gas molecules to the solid-liquid interface. These results suggest that the substrate with GML more easily attracts gas molecules from the bulk liquid. The potential well of -8.06 k_B_T indicates that the gas molecules in GML hardly diffuse into bulk water. The second local minima in the PMF curve that is located at distance of 0.63 nm for substrate with GML corresponds to the second gas layer, whose potential well is -4.15 k_B_T which is much smaller than that of the first layer (-8.06 k_B_T), indicating that the second gas layer is less stable than the first one, which is consistent with the findings of Lu et al. [32], that reports that the first gas layer on HOPG is stable while the second layer disappears with time. In addition to the ability to attract gas molecules from the bulk, the fluidity of gas molecules from GML to INB is also essential for the stability of INBs. Fig. 6a, b shows snapshots of the simulations at 10 and 20 ns, respectively. The green, red, and blue beads represent N_2_ molecules in GML, INB, and bulk liquid, respectively. It is clear that these gas molecules intermingled with each other after 10 ns. This indicates the fluidity of the gas molecules in GML, which guarantees that gas molecules attracted from the bulk liquid could flow into the INBs through the GML channel to compensate for the outward diffusion.

## 4 Discussion

This study explored the effect of GML on contact angle and stability of INBs. Once GML is formed, the solid-liquid interaction is weakened owing to the hindering of GML, while the solid-gas interfacial tension is largely reduced by the high gas density; hence, the contact angle is always much larger than its macroscopic counterpart regardless of the surface hydrophobicity, which is consistent with the experimental observations [8,[49], [50], [51]]. The contact angle of macrobubble is not affected by GML because GML can not coexist with macrobubbles because of the low gas density inside the bubble (Fig. 1). For hydrophilic substrates, INBs are found on mica surfaces, but rarely on quartz surfaces [8], which may be because the solid-gas interaction for mica is stronger than that of quartz. The experiments conducted by Arif et al. [55] and Sarmadivaleh et al. [56] showed that the advancing contact angle for mica at 308 K increased from 0° to approximately 110° when the CO_2_ pressure was increased from 0.1−20 MPa, while this variation was from 0° to approximately 32° for quartz at 296 *K*, indicating that the solid-gas interaction of mica might be much stronger than that of quartz. Therefore, strong solid-gas interactions might be an essential condition for the formation of INBs on hydrophilic substrates such as mica.

**Fig. 7:**
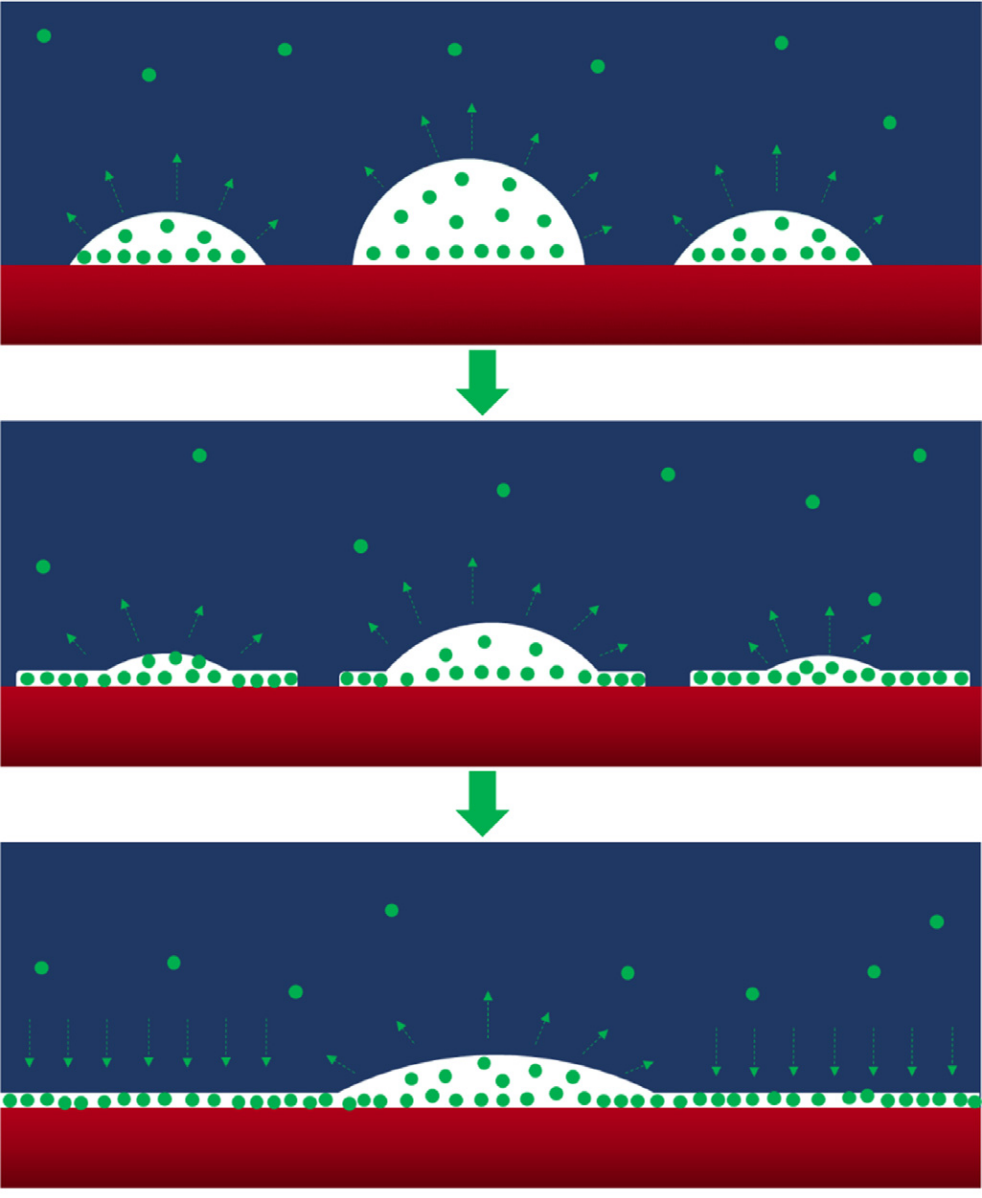
**Schematic diagram for formation and stability of INB**. Red color represents substrate; blue color represents liquid; and green beads represent gas molecules.

Based on the results of this study, we propose a formation process for INBs, as shown in Fig. 7. (ⅰ) The gas oversaturation caused by solvent exchange or other methods nucleates many micro-nano bubbles (forms directly on the substrate or from the attachment of bulk bubbles), these bubbles shrink due to the outward diffusion of gas molecules, and thus the internal gas density increases gradually with the shrinking bubble. (ⅱ) The GML begins to form when the internal gas density is sufficiently high. (ⅲ) The GML continues to grow because of the contribution from INBs and adsorption of gas molecules from the bulk liquid until the hydrophobic area is fully covered with GML; after which the remaining INBs stop shrinking and remain stable. In this process, if some INBs become too small, such that the inside gas density reaches a critical value, these INBs turn into GML. Hence, once the final stable INBs are formed, together with the sacrifice of many pioneer nanobubbles, the role of GML shifts from a nanobubble predator to a gas collector panel.

According to experimental observations, the coverage percentage of INBs is usually lower than 10% [8,15], that is, each INB has an area several times larger to attract gas molecules to compensate for outward diffusion, thus stabilizing INB. Lu et al. [35] reported that GML could exist in undersaturated water, which is a likely explanation for the fact that INBs are stable in undersaturated aqueous solutions [21]. In addition, contact-line pinning is not necessary in this theory. This is a dynamic equilibrium process, that only requires the intrinsic thermal motion of gas molecules, with no need for an extra energy source. The only precondition of this theory is the high gas density inside the bubble, because GML could not coexist with bubbles with low gas density. Zhou, et al. [29] demonstrated that the internal oxygen density is approximately 118 g/L, which is approximately 100 times larger than that of ambient air, while the calculated Laplace pressure is approximately 1.8 atm considering that the lateral diameter and height of this INB are approximately 540 nm and 46 nm, respectively. Although the ultra-high gas density inside INBs has been confirmed, further research is still needed to identify the mechanism for much higher gas density than predicted by Young-Laplace equation. Some experiments have also found that small cavities have much higher gas densities than those estimated by Young-Laplace equation [33,[57], [58], [59], [60]]. It is possible that the gas density inside INB with nanoscale height is more affected by solid-gas interaction than Laplace pressure, which needs further study.

## 5 Conclusion

In this study, the influence of gas density and GML on contact angle was studied through MD simulations, and the role of GML in the stability of INBs was also discussed. With the increase in gas density, contact angle of water was found to increase significantly first, which is mainly due to the decrease in solid-gas interfacial tension. When nitrogen density reached 2.57 nm^−3^, a GML was formed, after which the contact angle increased gently with gas density, which is in accordance with the experimental observations that contact angle of INB increased slightly with decrease in height. When nitrogen density reached as high as 11.38 nm^−3^, the contact angle was 180${}^{\circ }$, indicating that INBs would transform into GML when they were too small. Conversely, if the gas density was lower than 2.57 nm^−3^, the bubble would not coexist with GML, which seems to be consistent with the results that report that the INBs are always in a preferable size range of dozens to thousands of nanometers in lateral diameter. For substrates with vacuum contact angles of 35.71°, 65.89°, 90.30° and 106.72°, respectively, their contact angles under high gas density after the formation of GML were greater than 140°, which mirrors previous reports stating that the contact angle of INBs is usually very large regardless of surface hydrophobicity. Further investigations indicated that GML could only be formed on the substrate with $\varepsilon_{sg}>\varepsilon_{sl}$, but not on the substrate with $\varepsilon_{sg}<\varepsilon_{sl}$ even when the internal gas density is very high.

According to the PMF curves, the N_2_ molecule is attracted by the substrate from a distance of 0.54 nm for the bare substrate, while that for the substrate with GML is 1.18 nm. In addition, there are two potential barriers of 0.31 and 0.87 k_B_T due to steric effect from hydration shell when N_2_ molecules are diffusing towards the bare substrate; while there is almost no barrier for the substrate with GML. Hence, the substrate with GML more easily attracts gas molecules from the bulk liquid than the bare substrate, indicating the ability of GML to function as a gas reservoir. Further research showed that the gas molecules from GML, INB, and bulk intermingled with each other after 10 ns. This indicates the fluidity of gas molecules in GML, which confirms that the gas molecules attracted from the bulk liquid could flow into the INBs through GML channel to compensate for outward diffusion. Therefore, the formation of GML favors the stability of INBs.

Through this study, the formation of the GML justifies the large contact angle and long-term stability of INBs. An important precondition is the high gas density inside bubbles, which does not require gas oversaturation or contact line pinning. Although the ultrahigh gas density inside INBs has been confirmed through previous experiments, further research is still needed to explain the mechanism for a much higher gas density inside INBs than predicted by Young-Laplace equation.

## Declaration of competing interest

The authors declare that they have no conflicts of interest in this work.

## Appendix Supplementary materials

Image, application 1

## Footnotes

Supplementary material associated with this article can be found, in the online version, at doi:10.1016/j.fmre.2022.05.005.

## References

[1] Parker J.L., Claesson P.M., Attard P. (1994). Bubbles, cavities, and the long-ranged attraction between hydrophobic surfaces. J. Phys. Chem..

[2] Ljunggren S., Eriksson J.C. (1997). The lifetime of a colloid-sized gas bubble in water and the cause of the hydrophobic attraction. Colloids and Surfaces A.

[3] Lou S., Ouyang Z., Yi Z. (2000). Nanobubbles on solid surface imaged by atomic force microscopy. J. Vac. Sci. Technol. B.

[4] Ishida N., Inoue T., Miyahara M. (2000). Nano bubbles on a hydrophobic surface in water observed by tapping-mode atomic force microscopy. Langmuir.

[5] Zhang X., Khan A., Ducker W.A. (2007). A nanoscale gas state. Phys Rev Lett.

[6] Steitz R., Gutberlet T., Hauss T. (2003). Nanobubbles and their precursor layer at the interface of water against a hydrophobic substrate. Langmuir.

[7] Hampton M.A., Nguyen A.V. (2010). Nanobubbles and the nanobubble bridging capillary force. Advances in Colloid and Interface Science.

[8] Lohse D., Zhang X. (2015). Surface nanobubbles and nanodroplets. Reviews of Modern Physics.

[9] Alheshibri M., Qian J., Jehannin M. (2016). A history of nanobubbles. Langmuir.

[10] Tan B.H., An H., Ohl C.-D. (2021). Identifying surface-attached nanobubbles. Current Opinion in Colloid & Interface Science.

[11] Brenner M.P., Lohse D. (2008). Dynamic equilibrium mechanism for surface nanobubble stabilization. Phys Rev Lett.

[12] Yang S., Tsai P., Kooij E.S. (2009). Electrolytically generated nanobubbles on highly orientated pyrolytic graphite surfaces. Langmuir.

[13] Chan C.U., Ohl C.D. (2012). Total-internal-reflection-fluorescence microscopy for the study of nanobubble dynamics. Phys Rev Lett.

[14] Dietrich E., Zandvliet H.J., Lohse D. (2013). Particle tracking around surface nanobubbles. Journal of Physics Condensed Matter.

[15] Ducker W.A. (2009). Contact angle and stability of interfacial nanobubbles. Langmuir.

[16] Zhang X., Uddin M.H., Yang H. (2012). Effects of surfactants on the formation and the stability of interfacial nanobubbles. Langmuir.

[17] German S.R., Wu X., An H. (2014). Interfacial nanobubbles are leaky permeability of the gas water interface. ACS Nano.

[18] Lohse D., Zhang X. (2015). Pinning and gas oversaturation imply stable single surface nanobubbles. Physical Review E.

[19] Tan B.H., An H., Ohl C.D. (2017). Resolving the pinning force of nanobubbles with optical microscopy. Phys Rev Lett.

[20] Bull D.S., Nelson N., Konetski D. (2018). Contact line pinning is not required for nanobubble stability on copolymer brushes. Journal of Physical Chemistry Letters.

[21] Qian J., Craig V.S.J., Jehannin M. (2019). Long-term stability of surface nanobubbles in undersaturated aqueous solution. Langmuir.

[22] Tan B.H., An H., Ohl C.D. (2018). Surface nanobubbles are stabilized by hydrophobic attraction. Phys Rev Lett.

[23] Tan B.H., An H., Ohl C.-D. (2019). Stability, dynamics, and tolerance to undersaturation of surface nanobubbles. Physical Review Letters.

[24] Zhang X., Chan D.Y., Wang D. (2013). Stability of interfacial nanobubbles. Langmuir.

[25] Marmur A. (1997). Line tension and the intrinsic contact angle in solid–liquid–fluid systems. J. Colloid Interface Sci..

[26] Weijs J.H., Snoeijer J.H., Lohse D. (2012). Formation of surface nanobubbles and the universality of their contact angles: A molecular dynamics approach. Phys Rev Lett.

[27] Petsev N.D., Leal L.G., Shell M.S. (2020). Universal gas adsorption mechanism for flat nanobubble morphologies. Phys Rev Lett.

[28] Wang C., Li Z., Li J. (2008). High density gas state at water graphite interface studied by molecular dynamics simulation. Chinese Physics B.

[29] Zhou L., Wang X., Shin H.J. (2020). Ultrahigh density of gas molecules confined in surface nanobubbles in ambient water. J Am Chem Soc.

[30] Wang S., Zhou L., Wang X. (2019). Force spectroscopy revealed a high-gas-density state near the graphite substrate inside surface nanobubbles. Langmuir.

[31] Fang C.K., Ko H.C., Yang C.W. (2016). Nucleation processes of nanobubbles at a solid/water interface. Sci Rep.

[32] Lu Y.H., Yang C.W., Hwang I.S. (2014). Atomic force microscopy study of nitrogen molecule self-assembly at the HOPG–water interface. Appl. Surf. Sci..

[33] Lu Y.H., Yang C.W., Fang C.K. (2014). Interface-induced ordering of gas molecules confined in a small space. Sci. Rep..

[34] Schlesinger I., Sivan U. (2018). Three-dimensional characterization of layers of condensed gas molecules forming universally on hydrophobic surfaces. J Am Chem Soc.

[35] Lu Y.H., Yang C.W., Hwang I.S. (2012). Molecular layer of gaslike domains at a hydrophobic–water interface observed by frequency-modulation atomic force microscopy. Langmuir.

[36] Peng H., Hampton M.A., Nguyen A.V. (2013). Nanobubbles do not sit alone at the solid-liquid interface. Langmuir.

[37] Abraham M.J., Murtola T., Schulz R. (2015). GROMACS: High performance molecular simulations through multi-level parallelism from laptops to supercomputers. Softwarex.

[38] Humphrey W., Dalke A., Schulten K. (1996). VMD: Visual molecular dynamics. J. Mol. Graph..

[39] Peng H., Birkett G.R., Nguyen A.V. (2013). Origin of interfacial nanoscopic gaseous domains and formation of dense gas layer at hydrophobic solid-water interface. Langmuir.

[40] Peng H., Birkett G.R., Nguyen A.V. (2013). The impact of line tension on the contact angle of nanodroplets. Molecular Simulation.

[41] Kumar S., Rosenberg J.M., Bouzida D. (1992). THE weighted histogram analysis method for free-energy calculations on biomolecules. I. The method. Journal of Computational Chemistry.

[42] Schlesinger I., Sivan U. (2017). New information on the hydrophobic interaction revealed by frequency modulation AFM. Langmuir.

[43] Tortora M., Meloni S., Tan B.H. (2020). The interplay among gas, liquid and solid interactions determines the stability of surface nanobubbles. Nanoscale.

[44] Yi H.M., Weiruo S., Maruti B. (1991). Adsorption and diffusion of nitrogen, oxygen, argon, and methane in molecular sieve carbon at elevated pressures. Separations Technology.

[45] Yang J., Duan J., Fornasiero D. (2003). Very small bubble formation at the solid− water interface. Journal of Physical Chemistry B.

[46] Kameda N., Sogoshi N., Nakabayashi S. (2008). Nitrogen nanobubbles and butane nanodroplets at Si(100). Surface Science.

[47] Guo W., Shan H., Guan M. (2012). Investigation on nanobubbles on graphite substrate produced by the water–NaCl solution replacement. Surface Science.

[48] Epstein P.S., Plesset M.S. (1950). On the stability of gas bubbles in liquid-gas solutions. J. Chem. Phys..

[49] Craig V.S.J. (2011). Very small bubbles at surfaces—the nanobubble puzzle. Soft Matter.

[50] Seddon J.R., Lohse D. (2011). Nanobubbles and micropancakes: Gaseous domains on immersed substrates. J Phys Condens Matter.

[51] Seddon J.R., Lohse D., Ducker W.A. (2012). A deliberation on nanobubbles at surfaces and in bulk. Chemphyschem.

[52] Maheshwari S., van der Hoef M., Rodri Guez Rodri Guez J. (2018). Leakiness of pinned neighboring surface nanobubbles induced by strong gas-surface interaction. ACS Nano.

[53] Maheshwari S., van der Hoef M., Zhang X. (2016). Stability of surface nanobubbles: A molecular dynamics study. Langmuir.

[54] Langmuir I. (1918). The adsorption of gases on plane surfaces of glass, mica and platinum. Journal of the American Chemical Society.

[55] Arif M., Al-Yaseri A.Z., Barifcani A. (2016). Impact of pressure and temperature on CO2-brine-mica contact angles and CO2-brine interfacial tension: Implications for carbon geo-sequestration. Journal of Colloid and Interface Science.

[56] Sarmadivaleh M., Al-Yaseri A.Z., Iglauer S. (2015). Influence of temperature and pressure on quartz-water-CO(2) contact angle and CO(2)-water interfacial tension. J Colloid Interface Sci.

[57] Birtcher R.C., Donnelly S.E., Song M. (1999). Behavior of crystalline Xe nanoprecipitates during coalescence. Physical Review Letters.

[58] Donnelly S.E., Evans J.H. (1991).

[59] Donnelly S.E. (1985). The density and pressure of helium in bubbles in implanted metals: A critical review. Radiat. Effects.

[60] Johnson P.B., Thomson R.W., Mazey D.J. (1990). Large bubble-like features ordered on a macrolattice in helium-implanted gold. Nature.

