# Supplementary material for: Contact angle and stability of interfacial nanobubble supported by gas monolayer
Source: Fundam Res. 2022 May 14;4(1):35–42. doi: 10.1016/j.fmre.2022.05.005 (PMC11630698; doi:10.1016/j.fmre.2022.05.005)
Supplement: Supplementary file 1 [file mmc1.pdf]

Supporting Information

The Role of Gas Monolayer on Contact Angle and Stability of Interfacial Nanobubble

Haichang Yang[a,b], Yaowen Xing[a*], Fanfan Zhang[a,b], Xiahui Gui[a], Yijun Cao[a,c*]

[a] Chinese National Engineering Research Center of Coal Preparation and Purification, China University of Mining and Technology, Xuzhou 221116, Jiangsu, China

[b] School of Chemical Engineering and Technology, China University of Mining and Technology, Xuzhou 221116, Jiangsu, China

c Henan Province Industrial Technology Research Institution of Resources and Materials, Zhengzhou University, Zhengzhou 450001, China

1 Fitting using Petsev et al.'s model.

Petsev et al.[1] demonstrate that the adsorption of gas molecules at solid surface lowers the energy of solid-gas interface, and quantifies them by the following equation:

$$\gamma_{sg}^{*} = \gamma_{sg} - \frac{k_B T}{b}\ln\left(1 + K_{eq}^{A} P\right) \quad (S1)$$

where γ_{sg}^{*} and γ_{sg} are respectively the solid-gas interfacial tension at air pressure *P* and in vacuum, k_B is Boltzmann constant, T is temperature, b is the cross-sectional area of an adsorbing gas molecule, and K_{eq}^{A} is the equilibrium adsorption constant. We transformed this equation by replacing air pressure (*P*) with nitrogen density (*c*) to apply it to high gas density system:

$$\Delta\gamma_{sg} = \gamma_{sg} - \gamma_{sg}^{*} = \frac{k_B T}{b}\ln\left(1 + 3.72 * 10^{6} * K_{eq}^{A} * c\right) \quad (S2)$$

where *c* is the number density of nitrogen molecules (nm^{-3}). According to Young's equation, $\gamma_{sg} = \gamma_{lg} * cos\theta + \gamma_{sl}$, the left side of eq. (S2) could be calculated by the equation of

$$\Delta\gamma_{sg} = (\gamma_{lg}^{*}\cos\theta^{*} - \gamma_{lg}\cos\theta) + (\gamma_{sl}^{*} - \gamma_{sl}) \quad (S3)$$

where θ^{*}, γ_{lg}^{*} and γ_{sl}^{*} are respectively the contact angle, liquid-gas and solid-liquid interfacial tension at gas density of c, and θ, γ_{lg} and γ_{sl} are respectively the contact angle, liquid-gas and solid-liquid interfacial tension in vacuum. The second term in the right side, namely the change of γ_{sl} with increasing gas density, is negligible. The liquid-gas interfacial tension as a function of gas density is shown in Fig. S1(a). Hence, the reduced solid-gas interfacial tension ($\Delta\gamma_{sg}$) could be calculated by

using eq. (S3), as shown in Fig. S1(b). Here, only the data without GML (first regime, marked in black color) in Fig. 1(a) of the main text was used for calculation of $\Delta\gamma_{sg}$. Clearly, $\Delta\gamma_{sg}$ is significantly increased with the increase of gas density, indicating the solid-gas interfacial tension is greatly decreased with increasing gas density. The relationship between $\Delta\gamma_{sg}$ and gas density can be fitted well by eq. (S2) with b and K_{eq}^{A} as fitting variables. The goodness of fit to the experimental data is R^2=0.9995, indicating an excellent description of data in the first regime in Fig. 1(a) of the main text in terms of Petsev et al.'s theory, and we obtain b=19.25 $Å^2$ and K_{eq}^{A}=3.28*10^{-6} Pa^{-1}, which is within the actual range of K_{eq}^{A}=(1.0~5.0)*10^{-6} Pa^{-1} for N_2, O_2 and Ar adsorbing to graphene[1, 2].

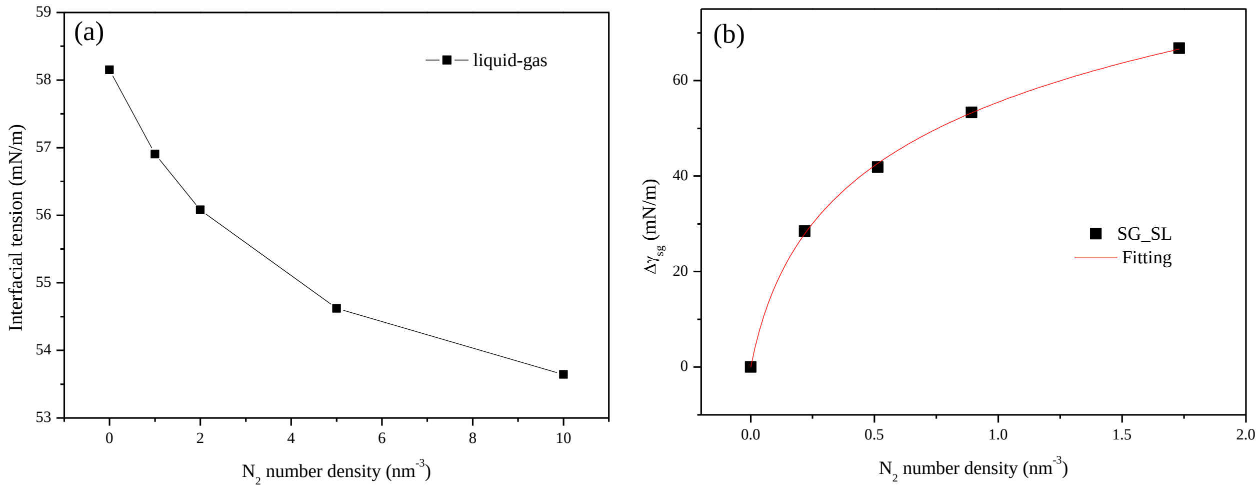

Fig. S1 (a) Liquid-gas interfacial tension as a function of gas density. (b) $\Delta\gamma_{sg}$ as a function of gas density, and the red solid line is fitting curve using eq. (S2).

2 Simulation Methodology for liquid-gas interfacial tension

The liquid-gas interfacial tension can be calculated from the anisotropy of the pressure tensor in x, y and z directions in MD simulation[3],

$$\gamma_{lg} = \frac{1}{2}L_z\left\langle P_{zz} - \frac{1}{2}\left(P_{xx} + P_{yy}\right)\right\rangle \qquad (S4)$$

where L_z is the length of the simulation box in z direction (perpendicular to the liquid-gas interface), and P_{xx}, P_{yy}, and P_{zz} are pressure tensors in x, y and z directions respectively. The snapshot of the liquid-gas interfacial tension simulation is shown in Fig. S2. The size of simulation box was 6.0*6.0*20.0 nm^3, while the thickness of water slab was around 6 nm. The simulation time was 20 ns, and the last 15 ns was used for calculating the interfacial tension.

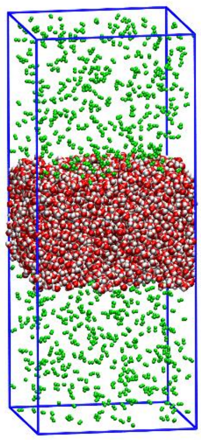

Fig. S2 Snapshots of simulation for liquid-gas interfacial tension. Green beads represent nitrogen atoms, and the red and white beads represent the oxygen and hydrogen atoms of water molecule.

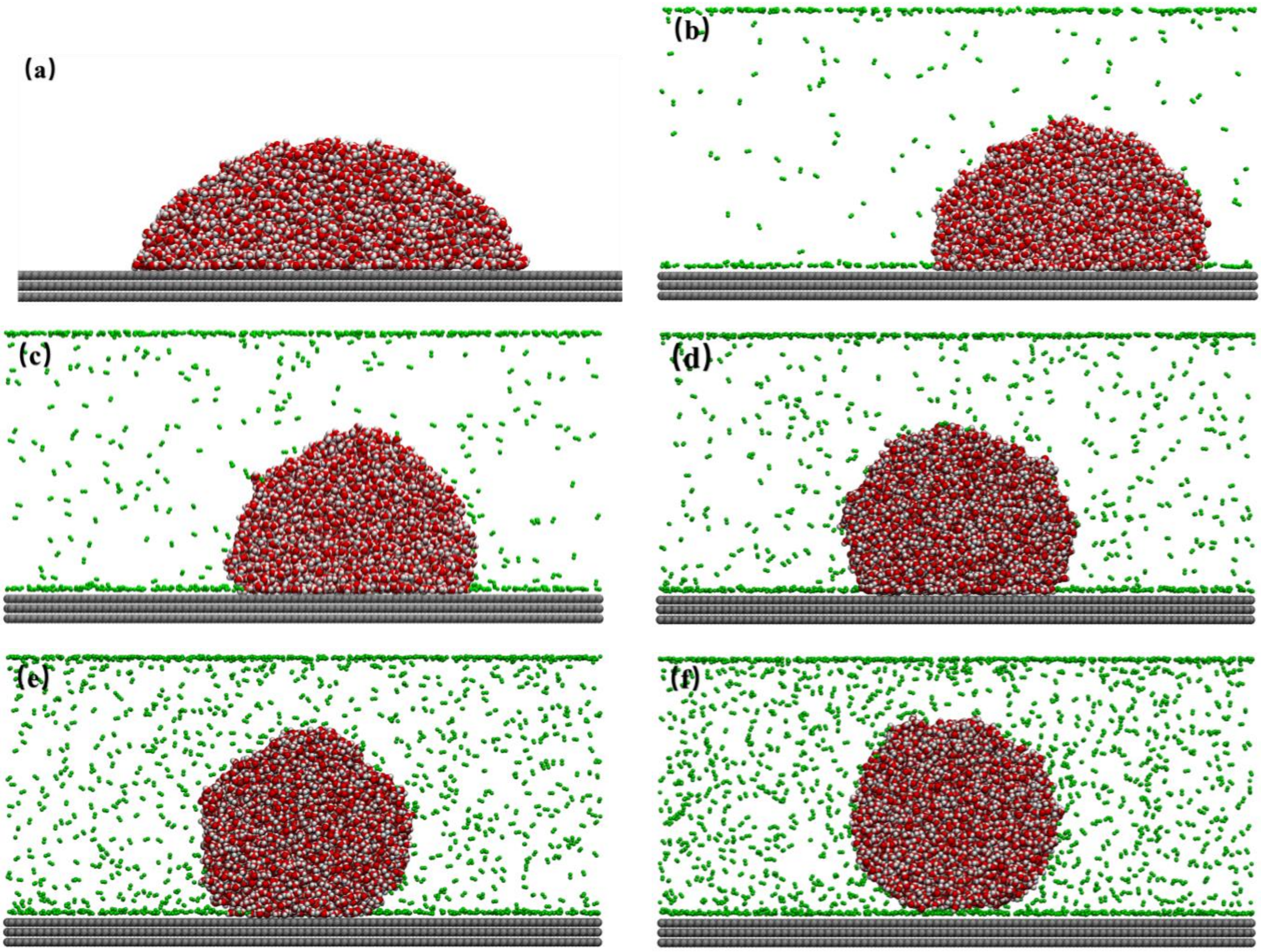

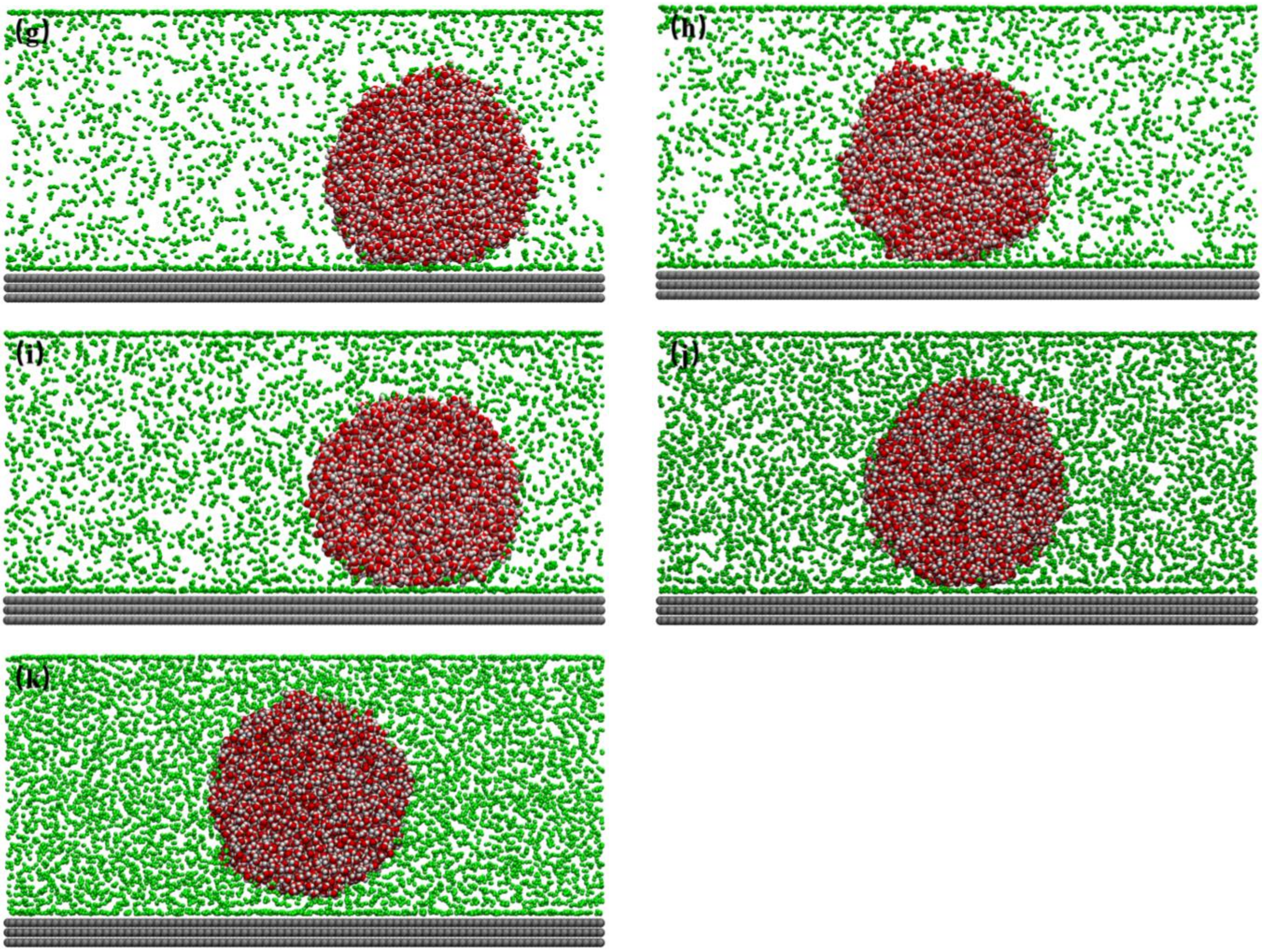

Fig. S3 Snapshots of each simulation in Fig. 1 of the main text. Figures (a)-(k) are corresponding to the increased gas density of 0, 0.22, 0.51, 0.89, 1.73, 2.57, 3.58, 4.61, 5.68, 8.92 and 11.38 nm^{-3}, respectively.

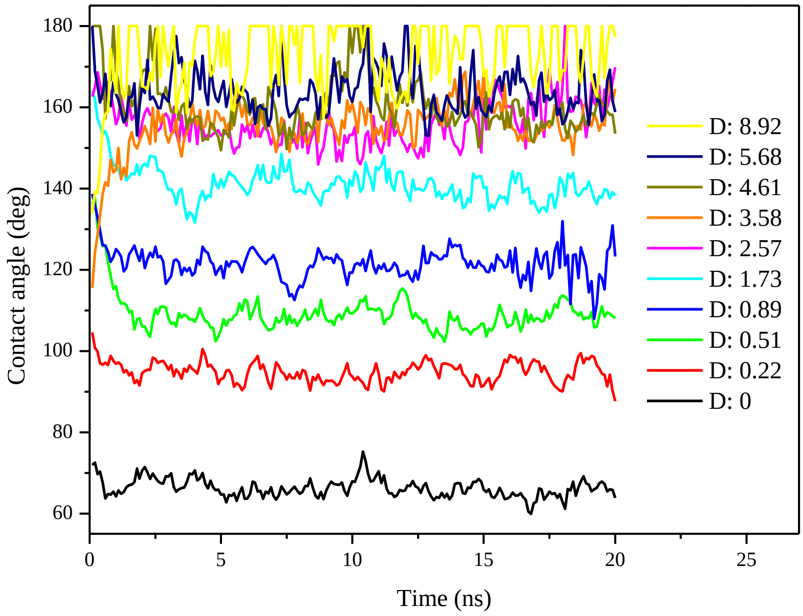

Fig. S4 Evolution of contact angle with time for simulation of different gas density in Fig. 1 of the main text. The

number of "D:" represents the gas density (nm^{-3}).

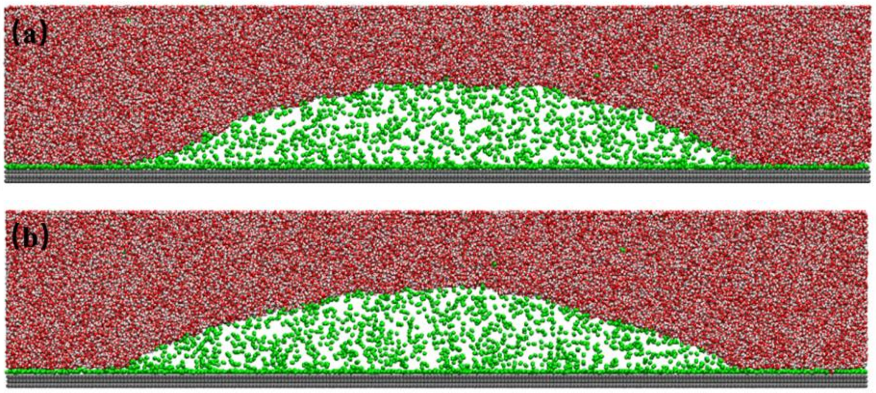

Fig. S5 The snapshots of INB formed on (a) SG_SL2 and (b) SG_SL3 substrates at 20 ns. These two simulations started with the configuration of Fig. 3(d) of the main text but using different solid-liquid interaction, and it was found the configuration kept stable during the 20 ns simulation. The contact angle as a function of time is shown in Fig. S6.

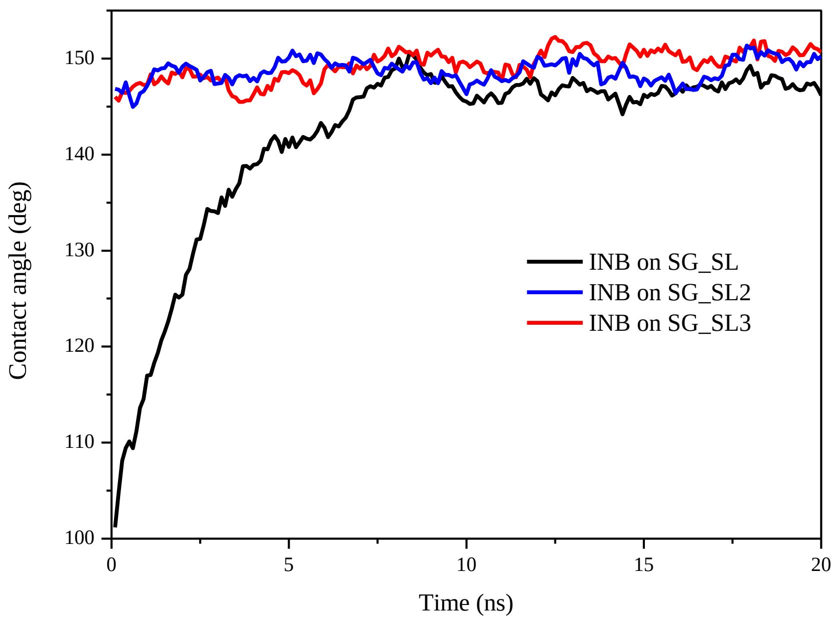

Fig. S6 The evolution of contact angle with time for INB on SG_SL, SG_SL2, and SG_SL3. The contact angles of water droplet in vacuum on these three substrates are 65.89°, 90.30° and 106.72° respectively

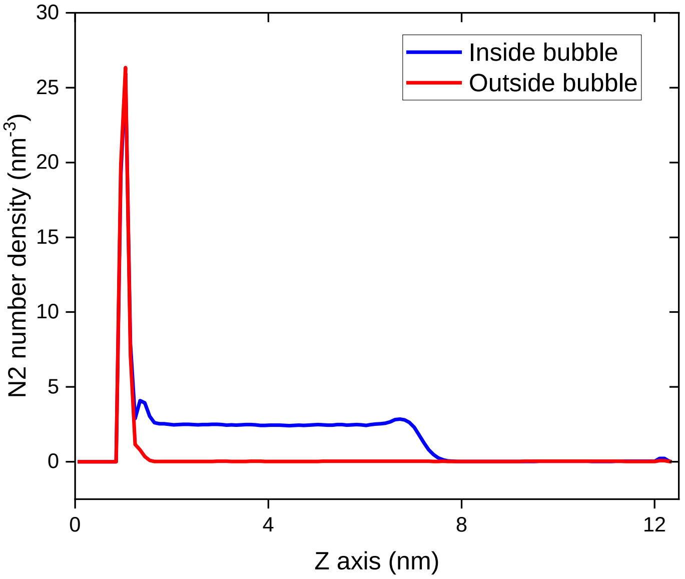

Fig. S7 The N_2 number density inside and outside bubble as a function of z axis.

REFERENCES

[1] N.D. Petsev, L.G. Leal, M.S. Shell, Universal Gas Adsorption Mechanism for Flat Nanobubble Morphologies, Phys. Rev. Lett. 125 (2020) 146101.

[2] H.M. Yi, S. Weiruo, B. Maruti, et al., Adsorption and diffusion of nitrogen, oxygen, argon, and methane in molecular sieve carbon at elevated pressures, Separations Technology 1 (1991) 90-98.

[3] H. Jiang, F. Muller-Plathe, A.Z. Panagiotopoulos, Contact angles from Young's equation in molecular dynamics simulations, J. Chem. Phys. 147 (2017) 084708.
